# Managing hyperthyroidism patients with unconventional therapy

**DOI:** 10.20945/2359-3997000000470

**Published:** 2022-04-28

Arch Endocrinol Metab. 2022;66(1):134

Where you read:

**REFERENCES**

1. Gamero JA, Ruiz VG, Ibarra JP. Use of unconventional antithyroid therapy in patients with thiamazole agranulocytosis in the context of the COVID-19 pandemic. Arch Endocrinol Metab. 2022;66(1):XX-XX.

Should read:

**REFERENCES**

1. Alvarez-Gamero JC, García-Ruiz VR, Paz-Ibarra JL. Use of unconventional antithyroid therapy in patients with thiamazole agranulocytosis in the context of the COVID-19 pandemic. Arch Endocrinol Metab. 2022;66(1):132-3.

